# Attitudes About Administrative Burdens for Beneficiaries and Dental Care Providers in Medicaid

**DOI:** 10.1111/jphd.70032

**Published:** 2026-01-29

**Authors:** Simon F. Haeder, Pamela Herd, Donald P. Moynihan

**Affiliations:** ^1^ Division of Health Services Management & Policy College of Public Health, The Ohio State University Columbus Ohio USA; ^2^ Ford School of Public Policy University of Michigan Ann Arbor Michigan USA

**Keywords:** administrative burdens, beneficiary burdens, Medicaid, oral health, oral health access, provider burdens

## Abstract

**Objectives:**

To assess public attitudes about a variety of burden‐reducing policies related to oral health services in the Medicaid program.

**Methods:**

We fielded a national survey (*N* = 5784) using Lucid from May 7 to 15, 2024. Respondents were queried whether they supported seven burden‐reducing policies for the Medicaid program focused on both beneficiaries and oral healthcare providers related to oral health services. The survey also contained an experiment that highlighted (1) the importance of dental care, (2) administrative burdens for beneficiaries, (3) dental challenges for children in poverty, (4) dental challenges for children of color, or (5) administrative burdens for dental providers. We relied on descriptive statistics as well as linear probability models to assess.

**Results:**

Overall, we found substantial support for reducing burden, ranging from 73.2% for referral requirements for physicians to a high of 92.1% for facilitating dentist credentialing. We found no differences across the various informational treatments. Analysis of the pooled data indicated that Americans were broadly supportive of reducing burdens for both beneficiaries and providers. We identified consistent differences based on ideology, racial resentment, racial resentment, empathy, awareness of disparities, burden tolerance, administrative capital, and connection to the Medicaid program.

**Conclusion:**

The American public broadly supports burden reductions for both beneficiaries and providers. More research is needed to assess public attitudes related to oral health services policies.

## 1 Introduction

Good oral health and care play a crucial role for the health and well‐being of individuals, with implications for chronic conditions, mental health, academic performance, and a quality of life [1, 2, 3, 4, 5, 6, 7]. Moreover, poor oral health has a number of secondary implications including those for employment and productivity [5, 6]. At the same time, lack of dental insurance is even more common in the United States as compared to traditional health insurance [8, 9], and expenditures dedicated to dental care only make up a small part of overall health spending [5, 10]. However, cost barriers to care are consistently higher for dental services compared to other healthcare services [9, 11]. As a result, the overall utilization of dental care has been declining among adults [9] while disparities and inequities in dental health even exceed those in general medical services [12]. Evidence indicates that dental care varies substantially by income, race and ethnicity, age, and insurance status [11] with corresponding implications for dental health [7, 10, 13].

The underinvestment in and neglect of dental health is also apparent in Medicaid, the joint federal and state program that helps cover medical costs for some people with limited income and resources [14, 15]. Importantly, the Medicaid dental benefits landscape varies substantially for children and adults [9, 11, 16]. States are mandated to cover dental benefits for children in Medicaid and the Children's Health Insurance Program, but states have the option to provide varying degrees of adult Medicaid dental benefits, with some states not providing any adult dental benefits at all [11, 16]. As a result, state Medicaid programs vary widely in terms of eligibility criteria as well as services provided to adult beneficiaries [5, 8, 17]. Even in states where dental services are provided, they are often under threat of cuts [8, 18, 19]. The cuts to the Medicaid program and limitations on funding imposed on states via the 2025 budget reconciliation are expected to further put pressure on states to eliminate or reduce existing dental benefits for adults [16, 20]. Recent research suggests that the implications of these reductions in benefits could impact more than 36 million adults and lead to additional costs to the U.S. healthcare system approaching $2 billion [16]. The precarious status of dental coverage by itself is concerning as existing evidence indicates that dental benefits are positively associated with increases in access and utilization [16, 21, 22, 23, 24, 25, 26, 27, 28, 29]. Moreover, expanding benefits in Medicaid has served to mitigate ethnic and racial disparities [21, 22, 30]. As a result, expanding dental benefits more broadly to Medicaid beneficiaries has been widely suggested [10, 19, 31].

However, having access to insurance coverage alone has shown to be insufficient as many other barriers to care are prevalent [32]. One crucial barrier in the Medicaid program is so‐called administrative burdens, the compliance, learning, and psychological costs associated with accessing public benefits [33]. Not only do these bureaucratic barriers impede benefit take‐up in the first place [34, 35, 36, 37, 38, 39, 40], but they also affect beneficiaries' ability to connect with providers when in need of care [41, 42, 43]. With regard to dental care, both patients and providers are confronted with a number of administrative burdens that impact patients' ability to access care. On the provider side, dentists and other providers often face substantial administrative barriers with regard to enrollment, certification, and credentialing in the Medicaid program [44, 45]. They often are also confronted with additional burdens to remain in compliance with program management requirements and particularly in dealing with prior authorizations, claims, and billing procedures [44, 45, 46, 47]. Evidence suggests that filing claims and billing procedures may be particularly burdensome for providers [46, 47]. Notably, administrative costs are particularly high in the Medicaid program as compared to other insurance markets [46, 47]. Importantly, administrative barriers have been found to be one of the primary drivers of declining participation in Medicaid for providers [46]. To make things worse, however, this additional complexity for Medicaid administrative processes occurs in a setting of often substantially lower reimbursement rates [47, 48]. Data indicate that dental care is particularly affected by Medicaid's substandard reimbursement rates, although these rates vary substantially by state [48]. At the same time, patients face a slew of burdens beginning with the enrollment into the Medicaid program [14, 15, 49, 50, 51]. However, these burdens are further compounded when seeking care, with beneficiaries often experiencing steep learning costs in how to navigate their insurance benefits [38, 52]. Importantly, beneficiaries are often also confronted with outdated and incorrect information about their benefits and networks [53, 54, 55, 56].

Given these burdens, it is unsurprising that the United States is estimated to spend one third of its healthcare dollars on administration, far in excess of other developed nations [47]. And while various coverage expansions via the Affordable Care Act have done much to increase insurance coverage rates in the United States, getting an insurance card is only the first step towards accessing appropriate and meaningful care in a timely manner [57]. There is substantial evidence that these access challenges are borne disproportionally by traditionally marginalized populations including those of lower socio‐economic status, racial and ethnic minorities, and those with disabilities [33, 38]. Importantly, government policies, deliberate or not, often reinforce and exacerbate these burdens and further increase disparities and inequities [33, 38, 57]. Importantly, frustrations with administrative burdens also have far‐reaching consequences beyond accessing care such as poorer mental health [58, 59, 60] as well as a variety of secondary effects [15, 38, 58, 59, 60, 61].

While existing research has extensively assessed public preferences about public assistance programs like Medicaid [14, 62], public attitudes about service delivery and patient and beneficiary experiences have presently received scant attention. This omission is crucial as these administrative details have important implications for the reach of public programs as well as the program experiences of beneficiaries and providers [63, 64]. And while our understanding of administrative burdens in general [33, 65, 66] as well as public attitudes about them has grown over time [49, 50, 67, 68, 69, 70], no previous studies have addressed how the American public thinks about administrative barriers related to oral health services, a crucial component of individuals' overall health [3, 71, 72]. That is, does the American public support reducing burdens for both beneficiaries and oral health providers in the Medicaid program? And does additional information about the benefits of oral healthcare or burdens experienced by beneficiaries or providers increase their support? Moreover, do attitudes differ for burdens focused on beneficiaries as compared to those focused on providers?

Expanding research and understanding along these lines offers an important contribution to both the academic literature as well as the broader policymaking process given the centrality of burdens in the politics of the U.S. safety net today [33, 38]. This particularly holds true given the current efforts to substantially alter the U.S. public safety net as well as reshape the Medicaid program under the Trump Administration, which includes efforts to increase administrative burdens [73, 74, 75]. Given that administrative burdens are frequently employed as low‐transparency and opaque tools to reduce access to public benefits [33, 38], understanding public opinion holds important potential to impact public policymaking. This particularly holds due to the important role that public attitudes can play in influencing public policy making [76, 77, 78] and because evidence suggests a “direct link between public opinion on the welfare state and policy outputs” (783) [79]. More generally, congruence between public attitudes and public policies remains a crucial underpinning of democratic society and governmental legitimacy [80, 81, 82, 83]. Moreover, assessments of public attitudes are particularly important given the crucial role that Medicaid can play in decreasing health disparities and augmenting the employability and life quality of beneficiaries. Ultimately, the political economy of administrative burdens has important implications for both Medicaid beneficiaries as well as dentists and ancillary professions. Crucially, there is also no research that compares how the public thinks about the relative distribution of burdens. Both providers and clients complain about the administrative burdens they encounter, especially in the domain of health; we offer evidence about how the public assesses such claims. Given these important implications, policy debates should be grounded in a sound assessment of public attitudes.

## 2 Methods

### 2.1 Data

To assess public attitudes about burden reductions for both Medicaid beneficiaries and oral healthcare providers, we fielded a survey from May 7 to 15, 2024, using Lucid (Appendix A, Supplementary Appendix B–D). Lucid is a reputable survey provider which relies on quota sampling based on respondent characteristics including age, race, gender, education, and income that align well with US Census demographic margins [84, 85]. Lucid, one of the world's largest marketplaces for survey panels and the largest in the United States, generally relies on a double‐opt in procedure where respondents first opt in to the panel and then opt into specific surveys (for additional details on Lucid, see Coppock and McClellan [85]). Recent research indicates the validity and representativeness of Lucid's data [84, 85]. We further improved sample representativeness by weighting the data (see Supplementary Appendix C). Of the 10,038 respondents who opted into the survey, 9339 (93%) consented, and 5784 respondents completed the survey (62%). To ensure data quality, Lucid utilized attention screeners which we augmented with an additional mid‐survey attention check (loss: 31%) and Qualtrics' fraud detection tools (loss 3%). Table 1 contains detailed demographic information on the respondents. Lucid was compensated at a rate of $1.50 per completed response. The study received approval from the Texas A&M University institutional review board (STUDY2024‐0264).

**TABLE 1:** Overview of unweighted and weighted demographic information for survey respondents.

Demographics	*N*	Unweighted	Weighted
Partisanship			
Democrat	2105	36.8%	36.0%
Independent	1711	29.9%	29.6%
Republican	1908	33.3%	34.4%
Ideology			
Liberal	1475	25.8%	24.7%
Moderate	2614	45.7%	45.0%
Conservative	1633	28.5%	30.3%
Insurance coverage			
Employer‐Sponsored Insurance	1495	36.8%	28.7%
Medicaid Coverage	1243	21.7%	18.8%
Medicare Coverage	1472	25.7%	28.1%
Individual Market	773	13.5%	12.3%
Uninsured	410	7.2%	6.5%
Medicaid connection			
Ever Been on Medicaid	5784	40.0%	35.5%
Used Medicaid Oral Health Services Before	5784	27.6%	25.4%
Children Used Medicaid Oral Health Services Before	5784	17.4%	16.8%
Other Family Member Used Medicaid Oral Health Services Before	5784	26.3%	26.1%
Sex	5784		
Female	3004	51.9%	51.5%
Race/Ethnicity	5784		
White	3888	67.2%	63.1%
Black	640	11.1%	12.9%
Asian	671	4.3%	3.0%
Hispanic	249	11.6%	16.7%
Income	5784		
Less than 14,000	832	14.4%	9.7%
15,000 to 24,999	723	12.5%	8.3%
25,000 to 34,999	694	12.0%	9.1%
35,000 to 49,999	829	14.3%	12.1%
50,000 to 74,999	1071	18.5%	15.8%
75,000 and over	1635	28.3%	45.0%
Education	5737		
Less Than High School	237	4.1%	11.9%
High School	1436	25.0%	27.8%
Some College	2156	37.6%	28.1%
College or More	1908	33.3%	32.2%
Age groups			
18 to 24 (reference)	768	13.3%	12.6%
25 to 44	2087	36.1%	33.9%
45 to 64	1986	34.3%	33.4%
65 and over	943	16.3%	20.1%
Self‐assessed health status	5721		
Poor (reference)	241	4.2%	3.7%
Fair	1279	22.4%	20.3%
Good	2402	42.0%	42.0%
Very Good	1336	23.4%	25.0%
Excellent	463	8.1%	9.1%
Racial resentment	3857		
Low	1323	34.3%	31.7%
Mid	1318	34.2%	34.4%
High	1216	31.5%	33.9%
Census region	5784		
Northeast	1127	19.5%	19.6%
Midwest	1100	19.0%	18.6%
South	2163	37.4%	37.4%
West	1394	24.1%	24.4%
State adult medicaid coverage	5781		
None	101	1.8%	1.6%
Emergency	1332	23.0%	23.6%
Limited	1106	19.1%	18.4%
Extensive	3242	56.1%	56.3%

*Note:* Data based on a survey of U.S. adults 5784 U.S. residents, May 7 to May 15, 2024.

### 2.2 Survey Structure

Before exposing respondents to the experiment, all respondents were provided with a brief introduction to the Medicaid program (Supplementary Appendix B). Subsequently, respondents were randomly assigned to one of six treatment groups or the control group (Supplementary Appendix B). While the control group was not provided with any additional information, we provided the other respondents with treatments highlighting (1) the implications of untreated oral health issues, (2) the role that administrative burdens play for Medicaid recipients seeking to access oral healthcare, (3) the connection between poverty and dental problems, (4) the connection between poverty and dental problems as well as racial/ethnic disparities, or (5) administrative burdens imposed on dental care providers in Medicaid (see Supplementary Appendix B for full description). For example, our information treatment focused on the implications of untreated oral health issues read as follows:

Untreated oral health issues can result in serious health complications that impact cardiac, respiratory, and mental health and increase the likelihood of emergency dental‐related hospitalizations. Poor oral health can also damage job opportunities and earnings. The importance of oral health is particularly pronounced for children. Oral health affects a child's ability to eat and speak properly, which directly impacts their nutrition and communication skills. Poor oral health in childhood may have long‐term consequences, as it has been linked to an increased risk of chronic diseases in adulthood, such as heart disease and diabetes.

Our pre‐registered expectation was that these informational treatments would increase support for burden reductions as compared to the control group by highlighting to respondents the importance of oral health services. We expect this to be a general priming effect but also offer new information to respondents as many Americans may not be aware of these challenges or the presence of disparities given the complexities of the U.S. healthcare system [86, 87, 88]. However, we note that because of the lack of findings in comparing the various treatments to the control group (described in detail below), we conducted a number of analyses on data that pooled all treatments, as well.

### 2.3 Outcome Variables

After being exposed to the informational treatments, respondents were queried whether or not they thought that state Medicaid programs should implement seven actual burden‐reducing policies related to oral health care (Supplementary Appendix D). This questioning is in line with previous research on burden reductions [50]. Two of these policies focused on burden reductions for beneficiaries not directly related to care including (1) the use of outreach campaigns and (2) ensuring that provider directories are accurate and updated regularly. We also asked respondents about two policies reducing burdens for patients by expanding the scope of practice for dental care extenders via (3) “direct access,” that is not requiring Medicaid enrollees to see a dentist first before getting certain services from a dental hygienist or (4) by allowing dental therapists to provide certain routine services. Extenders are healthcare professionals with advanced medical training that work in roles traditionally filled by the highest level of care providers such as physicians and dentists [89]. Often also referred to as advanced practice providers (APPs), these providers are intended to expand access by taking on lower levels of care and preserving higher level providers to focus on higher levels of care. In addition, we asked whether respondents favored burden reductions for dentists related to (5) credentialing and (6) reimbursement, two common complaints by providers [44, 45, 46, 47]. Lastly, we asked respondents about their support for (7) requiring physicians to refer children to dental providers if they do not already have one. For example, we asked respondents the following question:

Some states have made it easier for participating dentists to be reimbursed if they serve Medicaid clients. For example, states allow providers to use electronic billing to process claims.

In your opinion, what should states do?

- States should streamline claims submission to make it easier for participating dentists to be reimbursed if they serve Medicaid clients (1)
- States should not streamline claims submission to make it easier for participating dentists to be reimbursed if they serve Medicaid clients (2)

Both oral health providers and clients experience a number of burdens in the Medicaid program [14, 15, 38, 44, 45, 46, 47, 49, 50, 51, 52, 53, 54, 55, 56]. However, providers and Medicaid beneficiaries often differ in how they are perceived by society due to how perceptions of them are constructed socially [90]. That is, there is a substantial literature that has consistently shown that Medicaid beneficiaries are often viewed negatively and perceived as undeserving of public support [78, 91, 92, 93, 94]. And while these perceptions are frequently based on tropes and stereotypes, they nonetheless carry important implications for policymaking and public perceptions [90, 95, 96]. These negative framings generally do not extend to medical providers, including dentists, which are often socially constructed positively due to their educational attainment and role as healers [78, 90, 92, 93, 97, 98, 99]. Overall, our expectation was then that policies focused on reducing burdens for oral health providers would elicit higher levels of support as compared to those focused on Medicaid beneficiaries.

### 2.4 Subgroup Analyses

The literature on the welfare state as well as administrative burdens indicate that, in addition to assessing the outcome variables across all respondents, important insights can be gained by assessing a number of subgroups (Supplementary Appendix E). We hence assessed attitudes about oral and orthodontic services across a variety of common demographics including ideology (liberal, conservative) [15, 49, 50, 69, 70, 100, 101, 102, 103] and connection to the Medicaid program (ever been on Medicaid) [67, 70]. We also assessed differences based on respondents' level of racial resentment [49, 50, 67]. Here, we relied on the commonly used four‐question battery (each a 5‐point scale) that included such statements as Blacks have gotten less than they deserve, need to work their way up, need to try harder to be as well off as Whites, and that slavery and discrimination have made it more difficult for Blacks to be successful [104, 105]. This cumulative scale was divided into tertiles for ease of comparison.

We also relied on several measures from the administrative burden literature. This includes burden empathy, a 3‐question battery, which inquires whether respondents are concerned, feel compassion, or are moved by the experience of those most vulnerable to administrative burdens [49, 50], as well as administrative capital, a 5‐point measure that asks respondents about their ability to manage common administrative tasks like “renewing your driver's license, registering your car, or signing up for insurance.” In addition, we utilized a validated generic burden tolerance instrument made up of four individual questions not tied to a specific policy [69]. Lastly, we queried respondents about the degree (5‐point scale) to which they believed that burdens have a greater impact for Non‐Hispanic Whites versus persons of color [49, 50, 67].

### 2.5 Methods

We analyzed the binary outcome variables using weighted linear probability models to facilitate analysis and display of the data [106, 107]. We did so by estimating a series of linear probability models and then made comparisons of predicted means to assess statistical and substantive significance [108, 109]. These assessments serve as *t*‐tests in a weighted environment. Because we relied on probability weights in our analyses, Stata automatically employed the robust variance estimator (the sandwich formula or HC1). For subgroup analyses, we interacted indicators for the variables of interest with the treatment indicators. Weighted logit models and assessments of average marginal effects broadly confirmed these findings (omitted). We considered a *p* value lower than 0.05 as statistically significant throughout our analyses.

## 3 Results

### 3.1 Descriptive Findings

**FIGURE 1:**
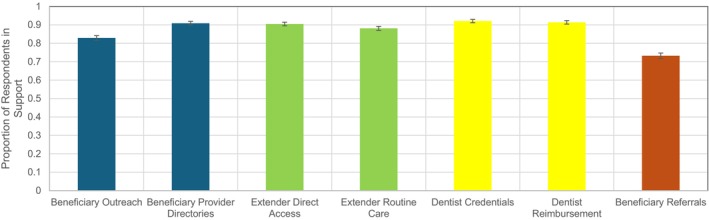
Respondents support for the implementation for various policies for all respondents, pooled across treatments. Based on a national survey of 5784 U.S. residents from May 7 to 15, 2024. Respondents were offered the binary choices of whether states should implement the burden‐reducing policies. Higher probabilities indicate higher levels of support for implementing the policy. For details on policy choices, refer to the text and the appendix. [Color figure can be viewed at wileyonlinelibrary.com]

**FIGURE 2:**
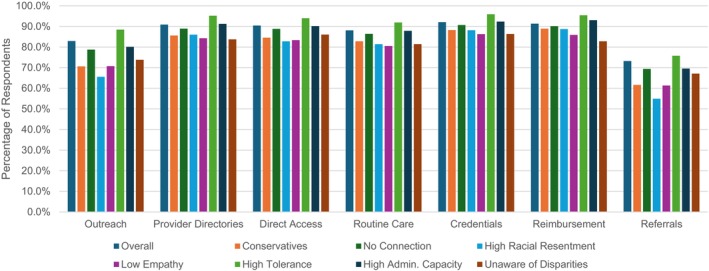
Respondents support for the implementation for various policies for various subgroups, pooled across treatments. Based on a national survey of 5784 U.S. residents from May 7 to 15, 2024. Respondents were offered the binary choices whether states should implement the burden‐reducing policies. Higher probabilities indicate higher levels of support for implementing the policy. For details on policy choices refer to text and the appendix. confidence bounds omitted for clarity. [Color figure can be viewed at wileyonlinelibrary.com]

Overall, we found that respondents overwhelmingly favored burden‐reducing policies in all seven cases we analyzed using the pooled data (Figure 1, Supplementary Appendix F). Across all respondents, support ranged from a low of 73.2% (95% CI: 71.8%–74.7%) for referral requirements for physicians to a high of 92.1% (91.2%–93.0%) for facilitating dentist credentialing. We also found strong support for Medicaid oral health services and lower tolerance for administrative burdens in such services among liberals, those with a connection to the Medicaid program, those low in racial resentment, those with high burden empathy, those with low burden tolerance generically, those with low administrative capital, and those aware of disparities associated with administrative burdens strongly favored burden‐reducing policies. For such groups, levels of support for Medicaid oral health services did not drop below 75% in any case (Supplementary Appendix F). However, even for demographics traditionally associated with lower levels of support for burden‐reducing policies (Figure 2, Supplementary Appendix F), support never dropped below the 54% mark. This included, for example, conservatives (61.6%–88.9%), those high in racial resentment (54.9%–88.7%), and those with low burden empathy (61.3%–86.3%). Thus, while there was significant variation across groups, the overall picture is one of broad support for oral health services and for making those services more accessible by reducing burdens.

### 3.2 Informational Effects

There is substantial evidence highlighting the importance of good dental health. However, it is also well‐established that Medicaid beneficiaries may struggle to access these services, and oral health providers face a number of burdens in offering these services. However, given the complexity of the U.S. healthcare system, many Americans may not be aware of these challenges or the presence of disparities, as noted above [86, 87, 88]. As noted above, we hence expected that providing respondents with information in these regards would increase their levels of support for the various burden‐reducing policies as compared to the control group. However, analyzing the experimental component of our data, we did not find any confirmation for our expectation for any of our treatments in any of the outcome variables we analyzed (Table 2). The small reduction in support for efforts to maintain provider directories in the oral health effects treatment serves as the sole exception (0.055, *p* = 0.003). As noted above, support across all policies and treatments was generally very high, raising the potential of ceiling effects for our treatments. That is, support for the burden‐reducing policies may already be so high that our informational treatments may not be able to substantially shift attitudes any further.

**TABLE 2:** Comparison of various treatments to control group across seven policies.

Policy	Treatment	Control predicted probability	Treatment predicted probability	Delta	*p*
Beneficiary outreach	Poor Oral Health Effects	0.815	0.819	−0.004	0.855
	Burdens on Beneficiaries	0.815	0.800	0.014	0.539
	Poverty	0.815	0.852	−0.037	0.088
	Poverty & Racial Disparities	0.815	0.849	−0.035	0.109
	Burdens on Providers	0.815	0.830	−0.016	0.499
Beneficiary provider directories	Poor Oral Health Effects	0.923	0.869	0.055	0.003
	Burdens on Beneficiaries	0.923	0.918	0.005	0.737
	Poverty	0.923	0.940	−0.017	0.244
	Poverty & Racial Disparities	0.923	0.901	0.023	0.182
	Burdens on Providers	0.923	0.900	0.024	0.163
Extender direct access	Poor Oral Health Effects	0.908	0.888	0.020	0.271
	Burdens on Beneficiaries	0.908	0.916	−0.008	0.621
	Poverty	0.908	0.924	−0.016	0.316
	Poverty & Racial Disparities	0.908	0.904	0.004	0.800
	Burdens on Providers	0.908	0.880	0.028	0.135
Extender routine care	Poor Oral Health Effects	0.879	0.866	0.013	0.502
	Burdens on Beneficiaries	0.879	0.874	0.005	0.808
	Poverty	0.879	0.884	−0.005	0.811
	Poverty & Racial Disparities	0.879	0.882	−0.003	0.869
	Burdens on Providers	0.879	0.887	−0.008	0.680
Dentist credentials	Poor Oral Health Effects	0.909	0.919	−0.010	0.542
	Burdens on Beneficiaries	0.909	0.928	−0.019	0.238
	Poverty	0.909	0.938	−0.029	0.065
	Poverty & Racial Disparities	0.909	0.916	−0.007	0.663
	Burdens on Providers	0.909	0.904	0.005	0.786
Dentist reimbursements	Poor Oral Health Effects	0.907	0.899	0.008	0.639
	Burdens on Beneficiaries	0.907	0.910	−0.003	0.886
	Poverty	0.907	0.928	−0.020	0.207
	Poverty & Racial Disparities	0.907	0.913	−0.005	0.749
	Burdens on Providers	0.907	0.919	−0.012	0.479
Beneficiary referrals	Poor Oral Health Effects	0.741	0.718	0.023	0.371
	Burdens on Beneficiaries	0.741	0.692	0.049	0.061
	Poverty	0.741	0.745	−0.005	0.852
	Poverty & Racial Disparities	0.741	0.742	−0.002	0.944
	Burdens on Providers	0.741	0.752	−0.011	0.652

*Note:* Based on a national survey of 5784 U.S. residents from May 7 to 15, 2024. Respondents were offered the binary choices whether states should implement the burden‐reducing policies. Higher probabilities indicate higher levels of support for implementing the policy. For details on policy choices refer to text and the appendix.

### 3.3 Beneficiary vs. Oral Health Professional Provider Burdens

As noted above, social constructions of beneficiaries and providers in the Medicaid program substantially differ and generally come down in favor of providers in terms of positive constructions [14, 15, 38, 44, 45, 46, 47, 49, 50, 51, 52, 53, 54, 55, 56, 78, 91, 92, 93, 94]. We therefore expected differences in how the public views burdens for each group. These expectations were partially confirmed using both pooled (Table 3) and unpooled data (Supplementary Appendix G). That is, based on the pooled data, respondents were more supportive of reducing burdens on dentists related to both credentialing (delta: 0.092, *p* < 0.001) and reimbursement (0.085, *p* < 0.001) as compared to outreach campaigns for beneficiaries. Conversely, respondents were less supportive of the referral requirement, which decreased burdens for beneficiaries while simultaneously increasing burdens for physicians (−0.096, *p* < 0.001). These findings were mirrored when comparing these three policies to the expansion of routine care via extenders (0.041, *p* < 0.001; 0.034, *p* < 0.001; −0.147, < 0.001). In other words, the public was largely not supportive of shifting burdens away from beneficiaries by asking providers to do more. At the same time, respondents did not differentiate between their support for updated and accurate provider directories and the two burdens focused on dentists. However, respondents were once again less supportive in comparison to the referral requirement (−0.177, *p* < 0.001). Lastly, respondents were more supportive of the easing of credentialing requirements for dentists as compared to the direct access policy (0.016, *p* = 0.019) and less supportive with regard to the referral requirement (−0.172, *p* < 0.001).

**TABLE 3:** Comparison across policies, pooled treatments.

Policy 1	Policy 2	Predicted Probability 1	Predicted Probability 2	Delta	*p*
Outreach	Credentials	0.828	0.919	0.092	0.000
	Reimbursement	0.828	0.913	0.085	0.000
	Referral	0.828	0.731	−0.096	0.000
Provider directories	Credentials	0.908	0.919	0.011	0.108
	Reimbursement	0.908	0.913	0.004	0.538
	Referral	0.908	0.731	−0.177	0.000
Direct care	Credentials	0.903	0.919	0.016	0.019
	Reimbursement	0.903	0.913	0.009	0.185
	Referral	0.903	0.731	−0.172	0.000
Routine care	Credentials	0.879	0.919	0.041	0.000
	Reimbursement	0.879	0.913	0.034	0.000
	Referral	0.879	0.731	−0.147	0.000

*Note:* Based on a national survey of 5784 U.S. residents from May 7 to 15, 2024. Respondents were offered the binary choices whether states should implement the burden‐reducing policies. Higher probabilities indicate higher levels of support for implementing the policy. For details on policy choices refer to text and the appendix.

### 3.4 Comparisons Across Subgroups

Lastly, as noted above, the existing literature on administrative burdens indicates often significant differences in burden tolerance across various demographic groups. In our analyses, differences across all policies were generally present in comparisons based on respondents' level of racial resentment, burden empathy, and their level of awareness of disparities associated with administrative burdens (Supplementary Appendices H–J). Overall, these factors consistently explained views about both burdens on beneficiaries and burdens on beneficiaries, suggesting that such factors shape a general worldview about making services both simpler to provide and to use. Liberals and conservatives differed from each other for all policies except dentist reimbursement (Supplementary Appendix K). The same held for comparing those with to those without connection to the Medicaid program (Supplementary Appendix L) while those with different levels of burden tolerance differed for all policies except the referral requirement (Supplementary Appendix M). Lastly, differences between respondents with different levels of administrative capital were only rarely present (Supplementary Appendix N).

## 4 Discussion

Good dental health is important for individuals' health and longevity. However, many Americans, even those with access to dental insurance, face substantial barriers to accessing appropriate care [10]. These barriers might be particularly common for Medicaid beneficiaries, many of whom do not have access to dental benefits in the first place [5, 8, 17]. However, even for those beneficiaries that do, administrative hurdles often create access challenges that are too substantial for many beneficiaries to overcome. Yet, patients and beneficiaries may not be the only participants in the Medicaid program experiencing such barriers, as medical providers such as dentists and ancillary professionals are consistently confronted with complex and burdensome administrative requirements [44, 45, 46, 47]. In many cases, these barriers may be deliberate policy tools imposed to reduce access [33, 38]. Importantly, these opaque barriers might be out of line with American public opinion and thus raise concerns about the democratic legitimacy of such policies. However, no research has previously evaluated this issue. Relying on a large‐scale survey, we assessed what the American public thinks about reducing administrative barriers to care for Medicaid beneficiaries.

Overall, our findings indicate overwhelming support for a number of burden‐reducing policies, and this support was present across a broad array of demographic groups. Given the high levels of support, we did not identify an effect for various informational treatments highlighting (1) the implications of untreated oral health issues, (2) the role that administrative burdens play for Medicaid recipients seeking to access oral healthcare, (3) the connection between poverty and dental problems, (4) the connection between poverty and dental problems as well as racial/ethnic disparities, or (5) administrative burdens imposed on dental care providers in Medicaid. However, we found that Americans are at times even more supportive of reducing administrative barriers for dentists that might impede their participation in the Medicaid program. Lastly, despite the overall high levels of support, we found consistent differences across different levels of ideology, racial resentment, empathy, awareness of disparities, burden tolerance, administrative capital, and connection to the Medicaid program.

This study is subject to a number of limitations. Substantively, respondents were only queried about certain burden‐reducing policies; attitudes may be different for others that we did not include in the survey. Moreover, we did not specifically focus on highlighting potential trade‐offs associated with burden reductions such as increased costs to the Medicaid program, although these are likely to be low [110], or implementation challenges. That is, public opinion may grow more negative if respondents are told that these policies will impose certain costs on the public. We believe that these effects are likely limited for two reasons. First, most respondents will realize that policy benefits are not free and require public monies. Second, as noted, the costs associated with Medicaid dental benefits are rather low. Nonetheless, given that no previous research exists, our findings hence offer an important baseline for future research that should explore these important nuances. Moreover, because of the dearth of knowledge on this topic, we also queried respondents broadly but provided sufficient policy details to allow them to make an informed decision. More intense treatments, including those offer additional policy details, could alter public perceptions. As in all surveys, specific wording choices or information presented in the treatments may also affect public attitudes. We again note that no prior research on this issue exists and that we included our survey questions in this manuscript as well as in the appendix. We also did not test specific information treatments that could have reduced support for the burden‐reducing policies. Future studies should address these limitations. Methodologically, we relied on non‐probability samples for our analyses, an approach common in survey research today [84, 85]. Moreover, we relied on a quality survey provider and took various additional steps to mitigate these limitations. Nonetheless, standard limitations of this approach apply.

Previous research health policy and access has been broadly neglectful of the important role that oral health has in general and oral health in Medicaid in particular [111, 112]. This lack of attention is concerning because it withholds crucial information and evidence from the policymaking process. Equally important, policies consistently out of line raise questions about democratic governance as well as government legitimacy and may lead to lower levels of trust and a decline in democratic norms [80, 81, 82, 83]. Overall, our findings here should be viewed as preliminary but invite more attention to the role of administrative burdens in oral health. While more work is needed to assess administrative barriers faced by both patients and providers, better understanding other factors decreasing access such as adequate provider participation is also of importance [5, 110, 113, 114, 115]. Meaningful insurance coverage requires that beneficiaries can access care when they need it. On the beneficiary side, this entails reducing barriers to accessing coverage and care. On the provider side, the calculation becomes more complex and may involve various policy decisions that affect provider experiences with Medicaid. Of course, provider decisions about program participation are complex and multi‐dimensional and providers must assess trade‐offs among a slew of variables including reimbursements and administrative burdens. Lastly, our findings here indicate broad support for improved access to oral health services in the Medicaid program. As policymakers consider relevant considerations such as cost and access, this should incorporate public preferences about burdens limiting both providers and users. Dentists and other dental professionals as well as dental associations should understand both the broad support for burden reduction, as well as variation in why some groups are more or less tolerant of burdens.

## Funding

Dr. Herd and Dr. Moynihan have received funding from the George E. Richmond Foundation.

## Conflicts of Interest

This research was declared exempt by the Texas A&M University institutional review board. Dr. Haeder has no disclosures to report related to this work. Dr. Herd and Dr. Moynihan have received funding from the George E. Richmond Foundation.

## Supporting information


**Data S1:** jphd70032‐sup‐0001‐Supinfo1.pdf.

## Data Availability

The data that support the findings of this study are available from the corresponding author upon reasonable request.

## Appendix A Survey Design

Our survey design included a survey experiment that, in addition to a control group, either highlighted (1) the importance of dental care, (2) administrative burdens for beneficiaries, (3) dental challenges for children in poverty, (4) dental challenges for children of color, or (5) administrative burdens for dental providers. We present these descriptions in detail in the appendix. However, extensive testing did not indicate statistically significant differences in our outcomes of interest. As a result, we present the pooled results in the manuscript.
