# Supplementary material for: Attitudes About Administrative Burdens for Beneficiaries and Dental Care Providers in Medicaid
Source: J Public Health Dent. 2026 Jan 29;86(1):47–59. doi: 10.1111/jphd.70032 (PMC12972262; doi:10.1111/jphd.70032)
Supplement: Supplementary file 1 — Data S1: jphd70032‐sup‐0001‐Supinfo1.pdf. [file JPHD-86-47-s001.pdf]

Appendix B

Treatments

Control

No treatment

Treatment 1

Untreated oral health issues can result in serious health complications that impact cardiac, respiratory, and mental health and increase the likelihood of emergency dental-related hospitalizations. Poor oral health can also damage job opportunities and earnings. The importance of oral health is particularly pronounced for children. Oral health affects a child's ability to eat and speak properly, which directly impacts their nutrition and communication skills. Poor oral health in childhood may have long-term consequences, as it has been linked to an increased risk of chronic diseases in adulthood, such as heart disease and diabetes.

Treatment 2

For enrollees, the administrative burdens of Medicaid can act as barriers to receiving dental care. Families need to apply for and maintain Medicaid coverage, which can include complex forms and documentation. Moreover, it's hard to find information about what services are covered and how to get them. It is also difficult to find dentists who will accept Medicaid coverage. Finally, many services require prior authorizations before Medicaid will pay for them, which adds another hoop that beneficiaries and their caregivers must jump through to get care. The difficulty accessing dental care helps explain why less than half of children covered by Medicaid received preventive dental services in 2018.

Treatment 3

Children in poor families are less likely to receive dental health care. Among children aged 12–19, 21% of children in poor families have untreated cavities compared to 11% of children in well-off families. At the same time, nearly twice as many poor adults have untreated cavities as do well-off adults.

Treatment 4

Children in families of color are less likely to receive dental health care. Among children aged 12–19, 20% of Black and Mexican American children have untreated cavities compared to 15% of White children. At the same time, nearly twice as many non-Hispanic Black or Mexican American adults have untreated cavities as do non-Hispanic White adults.

Treatment 5

For dental care providers, Medicaid's administrative burdens can make it harder to provide dental care. Providers must complete complex credentialing processes every few years, learn about and satisfy Medicaid administrative requirements, file large amounts of paperwork, and seek prior permission before providing some services. For many dental providers, the time and effort that they must spend to manage the administrative processes does not seem to be worth it, which partly explains a national shortage of dental care providers who accept Medicaid coverage.

Appendix C

Comparison of Raw and Weighted Qualtrics Data to National Benchmarks

Variable	Survey data (Raw)	Survey data (weighted)	Benchmark	Benchmark source
Female	52%	52%	51%	CPS
College degree	33%	32%	31%	CPS
Black	11%	13%	13%	CPS
White	67%	63%	62%	CPS
Hispanic	12%	17%	18%	CPS
Democrat	37%	36%	34%	ANES (Wgt.)
Republican	33%	34%	28%	ANES (Wgt.)
Mean age	46	48	47	ANES (Wgt.)
Median income	$35–49,999	$50–$74,999	$55–59,999	ANES (Wgt.)

Note: Comparison of the data to known population benchmarks. CPS = Current Population Survey. ANES = American National Election Study. Preference is given to CPS considering its sample size and representativeness, but make use of weighted ANES data whenever it was not possible to use CPS (i.e., CPS does not ask questions about Party ID). Weights in column two adjust for gender, education, race, age, and income. N (Survey Data) = 5784.

Appendix D

Outcomes

Next, we have a few questions about your views of the Medicaid program, the public insurance program that provides health insurance to low-income families and individuals. Medicaid is required to cover basic dental care for children, and states may decide to cover dental care for adults.

Some states use outreach strategies like mailing and calling campaigns to make it easier for Medicaid enrollees to find dental health providers and make appointments.

In your opinion, what should states do?

- States should engage in outreach strategies to help Medicaid enrollees find dental providers and make appointments (1)
- States should not engage in outreach strategies to help Medicaid enrollees find dental providers and make appointments (2)

Insurers' provider directories list dentists and other dental providers. Some states require these directories to be accurate and regularly updated. This makes it easier for Medicaid recipients to identify dental providers and schedule appointments.

In your opinion, what should states do?

- States should mandate that insurers keep Medicaid dental provider directories accurate and updated (1)
- States should NOT mandate that insurers keep Medicaid dental provider directories accurate and updated (2)

Some states have increased access to dental care by allowing for “direct access,” that is not requiring Medicaid enrollees to see a dentist first before getting certain services from a dental hygienist, such as cleanings. This reduces barriers to care and makes it easier for Medicaid enrollees to access these types of dental services.

In your opinion, what should states do?

- States should allow dental hygienists to provide Medicaid enrollees “direct access” to dental hygienists for certain services (1)
- States should NOT allow Medicaid enrollees to “direct access” dental hygienists for certain services (2)

Some states have moved to allow dental therapists to provide routine dental care, such as exams and fillings. As a result, Medicaid enrollees do not have to see a dentist first before getting certain routine dental services. This reduces barriers to care and makes it easier for Medicaid enrollees to access these types of dental services.

In your opinion, what should states do?

- States should allow dental therapists to provide these routine dental services for Medicaid enrollees (1)
- States should NOT allow dental therapists to provide these routine dental services for Medicaid enrollees (2)

Some states have worked to reduce burdensome administrative processes to make it easier for dentists to provide services to Medicaid beneficiaries. For example, states have made it easier to verify their credentials to be a Medicaid provider and simplified provider contracts.

In your opinion, what should states do?

- States should streamline administrative processes to make it easier for dentists to provide services to Medicaid enrollees (1)
- States should NOT streamline administrative processes to make it easier for dentists to provide services to Medicaid enrollees (2)

Some states have made it easier for participating dentists to be reimbursed if they serve Medicaid clients. For example, states allow providers to use electronic billing to process claims

In your opinion, what should states do?

- States should streamline claims submission to make it easier for participating dentists to be reimbursed if they serve Medicaid clients (1)
- States should not streamline claims submission to make it easier for participating dentists to be reimbursed if they serve Medicaid clients (2)

Some states require that physicians refer children to dental providers if they do not already have one. This reduces the difficulties for families on Medicaid to find a dental provider, but puts some additional requirements on physicians.

In your opinion, what should states do?

- States should mandate that physicians refer younger children to dental providers (1)
- States should NOT mandate that physicians refer younger children to dental providers (2)

Appendix E

Relevant Survey Questions

Ideology

We hear a lot of talk these days about liberals and conservatives. Here is a five-point scale on which the political views that people might hold are arranged from extremely liberal to extremely conservative. Where would you place yourself on this scale?

Extremely liberal

Liberal

Moderate; Middle of the road

Conservative

Extremely Conservative

Medicaid Connection

Do you know anyone who has ever been on Medicaid? Mark all that apply.

Myself

My spouse

My children

My parents

Other family member

A friend

Administrative Capital

In general, how difficult is it for you to complete such administrative tasks as renewing your driver‘s license, registering your car, or signing up for insurance?

Extremely difficult

Somewhat difficult

Neither easy nor difficult

Somewhat easy

Extremely easy

Knowledge of Disparities

To your knowledge, how are administrative burdens—the time and energy spent accessing and maintaining enrollment—in public assistance programs like Medicaid or food stamps impacting various social groups in the U.S.? Are administrative burdens.

Much worse on White people

Somewhat worse on White people

About equal

Somewhat worse on people of color

Much worse on people of color

Racial Resentment Battery

Please tell us the extent to which you agree or disagree with each of the following statements:

Over the past few years, Blacks have gotten less than they deserve.

Irish, Italian, Jewish, and many other minorities overcame prejudice and worked their way up. Blacks should do the same without any special favors.

It‘s really a matter of some people not trying hard enough; if Blacks would only try harder, they could be just as well off as whites.

Generations of slavery and discrimination have created conditions that make it difficult for Blacks to work their way out of the lower class.

All four items offer respondents a 5-point scale ranging from “Strongly Agree” to “Strongly Disagree” with a neutral “Neither agree nor disagree” option.

Empathy Battery

We developed the three questions to assess respondents‘ empathy towards individuals facing administrative burdens based on previous work by Pfattheicher et al. [116, 117] and Skinner-Dorkenoo et al. [118].

The three questions were:

I am very concerned about those most vulnerable to administrative burdens in public assistance programs like Medicaid or food stamps.

I feel compassion for those most vulnerable to administrative burdens in public assistance programs like Medicaid or food stamps.

I am quite moved by what can happen to those most vulnerable to administrative burdens in public assistance programs like Medicaid or food stamps.

Each question offered respondents a 5-scale ranging from “Strongly disagree” to “Strongly agree” with a neutral option of “Neither agree nor disagree.”

Burden Tolerance Battery

It is acceptable that people face some hassles when they are in contact with the government

Strongly disagree

Somewhat disagree

Neither agree nor disagree

Somewhat agree

Strongly agree

If people want to access public services and benefits, it is only fair that they have to make a significant effort to get them

Strongly disagree

Somewhat disagree

Neither agree nor disagree

Somewhat agree

Strongly agree

People should be responsible for figuring out how to access government services themselves; it is not the government‘s responsibility to help them

Strongly disagree

Somewhat disagree

Neither agree nor disagree.

Somewhat agree

Strongly agree

It is acceptable that people sometimes feel that it is difficult and time-consuming to apply for government services and benefits

Strongly disagree

Somewhat disagree

Neither agree nor disagree.

Somewhat agree

Strongly agree

Appendix F

Estimates of Support for Various Policies, by Subgroups

Subgroup	Policy	Point estimate	95% Confidence bounds	
Overall	Outreach	82.9%	81.6%	84.2%
	Referral	73.2%	71.8%	74.7%
	Provider Directories	90.9%	89.9%	91.9%
	Credentials	92.1%	91.2%	93.0%
	Reimbursement	91.4%	90.4%	92.3%
	Direct Care	90.5%	89.5%	91.4%
	Routine Care	88.1%	87.0%	89.2%
Liberals	Outreach	91.0%	89.1%	93.0%
	Referral	84.0%	81.6%	86.5%
	Provider Directories	93.8%	92.1%	95.6%
	Credentials	95.8%	94.4%	97.1%
	Reimbursement	94.0%	92.3%	95.8%
	Direct Care	94.5%	92.9%	96.2%
	Routine Care	92.1%	90.2%	93.9%
Conservatives	Outreach	70.6%	67.8%	73.5%
	Referral	61.6%	58.7%	64.6%
	Provider Directories	85.6%	83.4%	87.8%
	Credentials	88.2%	86.2%	90.2%
	Reimbursement	88.9%	87.0%	90.8%
	Direct Care	84.5%	82.3%	86.8%
	Routine Care	82.8%	80.5%	85.2%
No connection to Medicaid	Outreach	78.8%	77.1%	80.5%
	Referral	69.4%	67.5%	71.3%
	Provider Directories	88.9%	87.6%	90.2%
	Credentials	90.7%	89.5%	91.9%
	Reimbursement	90.1%	88.8%	91.4%
	Direct Care	88.8%	87.5%	90.1%
	Routine Care	86.4%	85.0%	87.8%
Connection to Medicaid	Outreach	90.4%	88.7%	92.0%
	Referral	80.1%	78.0%	82.3%
	Provider Directories	94.4%	93.0%	95.8%
	Credentials	94.6%	93.3%	95.9%
	Reimbursement	93.6%	92.2%	95.0%
	Direct Care	93.5%	92.0%	94.9%
	Routine Care	91.2%	89.5%	92.8%
Low racial resentment	Outreach	90.9%	88.9%	92.9%
	Referral	79.8%	77.1%	82.5%
	Provider Directories	96.4%	95.1%	97.7%
	Credentials	97.2%	96.3%	98.2%
	Reimbursement	96.8%	95.7%	97.9%
	Direct Care	95.0%	93.5%	96.5%
	Routine Care	94.2%	92.8%	95.7%
High racial resentment	Outreach	65.6%	62.2%	68.9%
	Referral	54.9%	51.5%	58.4%
	Provider Directories	86.0%	83.5%	88.5%
	Credentials	88.2%	85.9%	90.4%
	Reimbursement	88.7%	86.4%	91.0%
	Direct Care	82.8%	80.0%	85.5%
	Routine Care	81.4%	78.6%	84.2%
Low empathy	Outreach	70.7%	68.3%	73.2%
	Referral	61.3%	58.7%	63.9%
	Provider Directories	84.3%	82.3%	86.3%
	Credentials	86.3%	84.5%	88.1%
	Reimbursement	85.9%	84.0%	87.8%
	Direct Care	83.3%	81.3%	85.3%
	Routine Care	80.5%	78.4%	82.7%
High empathy	Outreach	93.4%	91.9%	95.0%
	Referral	84.8%	82.6%	86.9%
	Provider Directories	95.7%	94.2%	97.2%
	Credentials	95.7%	94.3%	97.2%
	Reimbursement	95.2%	93.8%	96.7%
	Direct Care	95.4%	94.0%	96.9%
	Routine Care	94.5%	93.0%	95.9%
Low burden tolerance	Outreach	88.5%	86.8%	90.2%
	Referral	75.8%	73.5%	78.0%
	Provider Directories	95.2%	94.0%	96.4%
	Credentials	95.9%	94.8%	97.0%
	Reimbursement	95.4%	94.2%	96.7%
	Direct Care	94.0%	92.6%	95.3%
	Routine Care	91.9%	90.4%	93.4%
High burden tolerance	Outreach	77.6%	75.2%	80.1%
	Referral	71.9%	69.2%	74.6%

Subgroup	Policy	Point estimate	95% Confidence bounds	
	Provider Directories	87.2%	85.1%	89.3%
	Credentials	88.7%	86.8%	90.6%
	Reimbursement	88.1%	86.1%	90.0%
	Direct Care	86.3%	84.2%	88.4%
	Routine Care	85.8%	83.7%	87.8%
Low admin. capital	Outreach	87.7%	85.4%	89.9%
	Referral	78.3%	75.5%	81.0%
	Provider Directories	92.4%	90.5%	94.4%
	Credentials	94.1%	92.6%	95.7%
	Reimbursement	92.6%	90.7%	94.4%
	Direct Care	93.0%	91.4%	94.7%
	Routine Care	91.3%	89.5%	93.2%
High admin. capital	Outreach	80.1%	78.1%	82.0%
	Referral	69.6%	67.4%	71.7%
	Provider Directories	91.2%	89.9%	92.6%
	Credentials	92.3%	91.1%	93.6%
	Reimbursement	93.0%	91.8%	94.2%
	Direct Care	90.2%	88.7%	91.6%
	Routine Care	87.9%	86.3%	89.4%
Unaware of disparities	Outreach	73.8%	69.5%	78.1%
	Referral	67.1%	62.7%	71.6%
	Provider Directories	83.8%	79.8%	87.7%
	Credentials	86.3%	82.9%	89.7%
	Reimbursement	82.8%	78.9%	86.7%
	Direct Care	86.0%	82.9%	89.2%
	Routine Care	81.4%	77.6%	85.3%
Aware of disparities	Outreach	89.6%	87.7%	91.5%
	Referral	79.9%	77.4%	82.4%
	Provider Directories	96.1%	94.8%	97.3%
	Credentials	95.2%	93.9%	96.6%
	Reimbursement	95.1%	93.7%	96.4%
	Direct Care	94.5%	93.0%	96.0%
	Routine Care	92.5%	90.8%	94.1%

Note: Based on a national survey of 5784 U.S. residents from May 7 to 15, 2024. Respondents were offered the binary choices whether states should implement the burden-reducing policies. Higher probabilities indicate higher levels of support for implementing the policy. For details on policy choices refer to text and the appendix.

Appendix G

Comparison Across Policies, by Treatment

Treatment	Policy 1	Policy 2	Predicted probability 1	Predicted probability 2	Delta	*p*	Policy 1
1	Outreach	Credentials	Outreach	0.815	0.909	0.094	0.000
		Reimbursement		0.815	0.907	0.093	0.000
		Referral		0.815	0.741	−0.074	0.002
	Provider Directories	Credentials	Provider Directories	0.923	0.909	−0.014	0.402
		Reimbursement		0.923	0.907	−0.016	0.339
		Referral		0.923	0.741	−0.183	0.000
	Direct Care	Credentials	Direct Care	0.908	0.909	0.001	0.951
		Reimbursement		0.908	0.907	−0.001	0.968
		Referral		0.908	0.741	−0.168	0.000
	Routine Care	Credentials	Routine Care	0.879	0.909	0.030	0.103
		Reimbursement		0.879	0.907	0.028	0.122
		Referral		0.879	0.741	−0.138	0.000
2	Outreach	Credentials	Outreach	0.819	0.919	0.100	0.000
		Reimbursement		0.819	0.899	0.080	0.000
		Referral		0.819	0.718	−0.101	0.000
	Provider Directories	Credentials	Provider Directories	0.869	0.919	0.051	0.006
		Reimbursement		0.869	0.899	0.030	0.123
		Referral		0.869	0.718	−0.151	0.000
	Direct Care	Credentials	Direct Care	0.888	0.919	0.031	0.070
		Reimbursement		0.888	0.899	0.011	0.557
		Referral		0.888	0.718	−0.170	0.000
	Routine Care	Credentials	Routine Care	0.866	0.919	0.054	0.003
		Reimbursement		0.866	0.899	0.033	0.084
		Referral		0.866	0.718	−0.148	0.000
3	Outreach	Credentials	Outreach	0.800	0.928	0.128	0.000
		Reimbursement		0.800	0.910	0.110	0.000
		Referral		0.800	0.692	−0.108	0.000
	Provider Directories	Credentials	Provider Directories	0.918	0.928	0.010	0.510
		Reimbursement		0.918	0.910	−0.008	0.644
		Referral		0.918	0.692	−0.226	0.000
	Direct Care	Credentials	Direct Care	0.916	0.928	0.012	0.421
		Reimbursement		0.916	0.910	−0.006	0.700
		Referral		0.916	0.692	−0.224	0.000
	Routine Care	Credentials	Routine Care	0.874	0.928	0.054	0.001
		Reimbursement		0.874	0.910	0.036	0.053
		Referral		0.874	0.692	−0.182	0.000

Treatment	Policy 1	Policy 2	Predicted probability 1	Predicted probability 2	Delta	*p*	Policy 1
4	Outreach	Credentials	Outreach	0.852	0.938	0.086	0.000
		Reimbursement		0.852	0.928	0.076	0.000
		Referral		0.852	0.745	−0.107	0.000
	Provider Directories	Credentials	Provider Directories	0.940	0.938	−0.002	0.850
		Reimbursement		0.940	0.928	−0.012	0.381
		Referral		0.940	0.745	−0.195	0.000
	Direct Care	Credentials	Direct Care	0.924	0.938	0.014	0.318
		Reimbursement		0.924	0.928	0.004	0.801
		Referral		0.924	0.745	−0.179	0.000
	Routine Care	Credentials	Routine Care	0.884	0.938	0.054	0.001
		Reimbursement		0.884	0.928	0.044	0.008
		Referral		0.884	0.745	−0.138	0.000
5	Outreach	Credentials	Outreach	0.849	0.916	0.067	0.000
		Reimbursement		0.849	0.913	0.063	0.000
		Referral		0.849	0.742	−0.107	0.000
	Provider Directories	Credentials	Provider Directories	0.901	0.916	0.016	0.349
		Reimbursement		0.901	0.913	0.012	0.479
		Referral		0.901	0.742	−0.158	0.000
	Direct Care	Credentials	Direct Care	0.904	0.916	0.013	0.431
		Reimbursement		0.904	0.913	0.009	0.582
		Referral		0.904	0.742	−0.162	0.000
	Routine Care	Credentials	Routine Care	0.882	0.916	0.034	0.041
		Reimbursement		0.882	0.913	0.031	0.071
		Referral		0.882	0.742	−0.140	0.000
6	Outreach	Credentials	Outreach	0.830	0.904	0.074	0.000
		Reimbursement		0.830	0.919	0.089	0.000
		Referral		0.830	0.752	−0.079	0.001
	Provider Directories	Credentials	Provider Directories	0.900	0.904	0.005	0.796
		Reimbursement		0.900	0.919	0.019	0.246
		Referral		0.900	0.752	−0.148	0.000
	Direct Care	Credentials	Direct Care	0.880	0.904	0.024	0.198
		Reimbursement		0.880	0.919	0.039	0.027
		Referral		0.880	0.752	−0.128	0.000
	Routine Care	Credentials	Routine Care	0.887	0.904	0.018	0.336
		Reimbursement		0.887	0.919	0.032	0.058
		Referral		0.887	0.752	−0.135	0.000

Note: Based on a national survey of 5784 U.S. residents from May 7 to 15, 2024. Respondents were offered the binary choices whether states should implement the burden-reducing policies. Higher probabilities indicate higher levels of support for implementing the policy. For details on policy choices refer to text and the appendix.

Appendix H

Comparison Across Levels of Racial Resentment

Policy	Treatment	Low racial resentment	High racial resentment	Delta	*p*
Outreach	Control	0.882	0.634	0.248	0.000
	Poor Oral Health Effects	0.896	0.655	0.241	0.000
	Burdens on Beneficiaries	0.889	0.560	0.328	0.000
	Poverty	0.943	0.725	0.219	0.000
	Poverty & Racial Disparities	0.919	0.695	0.224	0.000
	Burdens on Providers	0.913	0.657	0.255	0.000
Provider directories	Control	0.962	0.862	0.100	0.004
	Poor Oral Health Effects	0.936	0.822	0.115	0.005
	Burdens on Beneficiaries	0.960	0.846	0.114	0.002
	Poverty	0.984	0.918	0.065	0.003
	Poverty & Racial Disparities	0.947	0.866	0.081	0.012
	Burdens on Providers	0.980	0.848	0.132	0.000
Direct care	Control	0.948	0.849	0.099	0.012
	Poor Oral Health Effects	0.962	0.780	0.182	0.000
	Burdens on Beneficiaries	0.955	0.856	0.099	0.003
	Poverty	0.947	0.877	0.070	0.028
	Poverty & Racial Disparities	0.941	0.844	0.098	0.008
	Burdens on Providers	0.941	0.755	0.187	0.000
Routine care	Control	0.942	0.803	0.139	0.000
	Poor Oral Health Effects	0.958	0.791	0.166	0.000
	Burdens on Beneficiaries	0.932	0.833	0.100	0.007
	Poverty	0.919	0.809	0.110	0.005
	Poverty & Racial Disparities	0.927	0.827	0.101	0.008
	Burdens on Providers	0.949	0.801	0.149	0.000
Credentials	Control	0.968	0.844	0.124	0.001
	Poor Oral Health Effects	0.977	0.857	0.120	0.000
	Burdens on Beneficiaries	0.955	0.891	0.064	0.046
	Poverty	0.978	0.934	0.044	0.032
	Poverty & Racial Disparities	0.972	0.897	0.075	0.003
	Burdens on Providers	0.972	0.874	0.099	0.001
Reimbursement	Control	0.970	0.890	0.080	0.012
	Poor Oral Health Effects	0.979	0.848	0.130	0.000
	Burdens on Beneficiaries	0.942	0.917	0.025	0.454
	Poverty	0.970	0.916	0.054	0.031
	Poverty & Racial Disparities	0.977	0.862	0.116	0.000
	Burdens on Providers	0.966	0.877	0.089	0.006

Policy	Treatment	Low racial resentment	High racial resentment	Delta	*p*
Referral	Control	0.737	0.572	0.165	0.005
	Poor Oral Health Effects	0.762	0.544	0.219	0.000
	Burdens on Beneficiaries	0.778	0.516	0.262	0.000
	Poverty	0.840	0.531	0.309	0.000
	Poverty & Racial Disparities	0.776	0.545	0.231	0.000
	Burdens on Providers	0.885	0.599	0.286	0.000

Note: Based on a national survey of 5784 U.S. residents from May 7 to 15, 2024. Respondents were offered the binary choices whether states should implement the burden-reducing policies. Higher probabilities indicate higher levels of support for implementing the policy. For details on policy choices refer to text and the appendix.

Appendix I

Comparison Across Levels of Empathy

Policy	Treatment	Low racial resentment	High racial resentment	Delta	*p*
Outreach	Control	0.679	0.944	−0.265	0.000
	Poor Oral Health Effects	0.706	0.907	−0.201	0.000
	Burdens on Beneficiaries	0.684	0.930	−0.246	0.000
	Poverty	0.765	0.930	−0.164	0.000
	Poverty & Racial Disparities	0.712	0.941	−0.229	0.000
	Burdens on Providers	0.713	0.944	−0.231	0.000
Provider directories	Control	0.853	0.982	−0.129	0.000
	Poor Oral Health Effects	0.801	0.906	−0.105	0.009
	Burdens on Beneficiaries	0.897	0.945	−0.048	0.087
	Poverty	0.868	0.977	−0.108	0.000
	Poverty & Racial Disparities	0.807	0.962	−0.155	0.000
	Burdens on Providers	0.843	0.949	−0.107	0.000
Direct care	Control	0.836	0.961	−0.125	0.000
	Poor Oral Health Effects	0.812	0.925	−0.114	0.001
	Burdens on Beneficiaries	0.864	0.958	−0.095	0.000
	Poverty	0.860	0.964	−0.103	0.000
	Poverty & Racial Disparities	0.827	0.967	−0.141	0.000
	Burdens on Providers	0.809	0.936	−0.127	0.000
Routine care	Control	0.783	0.935	−0.152	0.000
	Poor Oral Health Effects	0.791	0.914	−0.123	0.001
	Burdens on Beneficiaries	0.796	0.952	−0.156	0.000
	Poverty	0.802	0.939	−0.137	0.000
	Poverty & Racial Disparities	0.812	0.968	−0.156	0.000
	Burdens on Providers	0.829	0.947	−0.118	0.000

Policy	Treatment	Low racial resentment	High racial resentment	Delta	*p*
Credentials	Control	0.828	0.962	−0.134	0.000
	Poor Oral Health Effects	0.880	0.933	−0.053	0.099
	Burdens on Beneficiaries	0.882	0.946	−0.064	0.013
	Poverty	0.885	0.961	−0.076	0.003
	Poverty & Racial Disparities	0.830	0.986	−0.156	0.000
	Burdens on Providers	0.861	0.934	−0.073	0.020
Reimbursement	Control	0.856	0.945	−0.089	0.004
	Poor Oral Health Effects	0.846	0.930	−0.083	0.016
	Burdens on Beneficiaries	0.857	0.950	−0.093	0.001
	Poverty	0.882	0.945	−0.063	0.020
	Poverty & Racial Disparities	0.830	0.967	−0.137	0.000
	Burdens on Providers	0.864	0.977	−0.113	0.000
Referral	Control	0.629	0.847	−0.218	0.000
	Poor Oral Health Effects	0.615	0.802	−0.187	0.000
	Burdens on Beneficiaries	0.576	0.790	−0.214	0.000
	Poverty	0.648	0.863	−0.215	0.000
	Poverty & Racial Disparities	0.605	0.883	−0.277	0.000
	Burdens on Providers	0.624	0.876	−0.252	0.000

Note: Based on a national survey of 5784 U.S. residents from May 7 to 15, 2024. Respondents were offered the binary choices whether states should implement the burden-reducing policies. Higher probabilities indicate higher levels of support for implementing the policy. For details on policy choices refer to text and the appendix.

Appendix J

Comparison Across Levels of Awareness of Disparities

Policy	Treatment	Low racial resentment	High racial resentment	Delta	*p*
Outreach	Control	0.710	0.868	−0.159	0.026
	Poor Oral Health Effects	0.741	0.870	−0.129	0.027
	Burdens on Beneficiaries	0.653	0.893	−0.240	0.000
	Poverty	0.763	0.940	−0.177	0.001
	Poverty & Racial Disparities	0.818	0.910	−0.091	0.049
	Burdens on Providers	0.702	0.896	−0.194	0.001
Provider directories	Control	0.835	0.962	−0.126	0.029
	Poor Oral Health Effects	0.798	0.927	−0.129	0.026
	Burdens on Beneficiaries	0.854	0.957	−0.103	0.028
	Poverty	0.930	0.986	−0.055	0.038
	Poverty & Racial Disparities	0.767	0.966	−0.200	0.000
	Burdens on Providers	0.871	0.941	−0.070	0.145

Policy	Treatment	Low racial resentment	High racial resentment	Delta	*p*
Direct care	Control	0.838	0.962	−0.124	0.019
	Poor Oral Health Effects	0.875	0.918	−0.043	0.301
	Burdens on Beneficiaries	0.897	0.972	−0.075	0.023
	Poverty	0.882	0.947	−0.065	0.104
	Poverty & Racial Disparities	0.853	0.949	−0.096	0.019
	Burdens on Providers	0.809	0.911	−0.102	0.045
Routine care	Control	0.819	0.938	−0.119	0.024
	Poor Oral Health Effects	0.816	0.902	−0.086	0.109
	Burdens on Beneficiaries	0.831	0.939	−0.108	0.012
	Poverty	0.830	0.921	−0.091	0.050
	Poverty & Racial Disparities	0.790	0.920	−0.130	0.013
	Burdens on Providers	0.809	0.914	−0.105	0.044
Credentials	Control	0.820	0.961	−0.141	0.016
	Poor Oral Health Effects	0.881	0.941	−0.060	0.137
	Burdens on Beneficiaries	0.894	0.958	−0.063	0.081
	Poverty	0.954	0.947	0.006	0.803
	Poverty & Racial Disparities	0.838	0.959	−0.121	0.005
	Burdens on Providers	0.813	0.930	−0.117	0.028
Reimbursement	Control	0.831	0.946	−0.115	0.029
	Poor Oral Health Effects	0.824	0.953	−0.130	0.017
	Burdens on Beneficiaries	0.857	0.946	−0.090	0.053
	Poverty	0.868	0.937	−0.070	0.147
	Poverty & Racial Disparities	0.805	0.950	−0.144	0.003
	Burdens on Providers	0.786	0.966	−0.180	0.001
Referral	Control	0.705	0.797	−0.092	0.162
	Poor Oral Health Effects	0.699	0.774	−0.075	0.236
	Burdens on Beneficiaries	0.609	0.761	−0.152	0.020
	Poverty	0.617	0.878	−0.261	0.000
	Poverty & Racial Disparities	0.696	0.788	−0.092	0.112
	Burdens on Providers	0.623	0.818	−0.195	0.002

Note: Based on a national survey of 5784 U.S. residents from May 7 to 15, 2024. Respondents were offered the binary choices whether states should implement the burden-reducing policies. Higher probabilities indicate higher levels of support for implementing the policy. For details on policy choices refer to text and the appendix.

Appendix K

Comparison Across Levels of Ideology

Policy	Treatment	Low racial resentment	High racial resentment	Delta	*p*
Outreach	Control	0.869	0.673	0.197	0.000
	Poor Oral Health Effects	0.919	0.689	0.230	0.000
	Burdens on Beneficiaries	0.934	0.620	0.314	0.000
	Poverty	0.910	0.786	0.124	0.001
	Poverty & Racial Disparities	0.886	0.792	0.094	0.016
	Burdens on Providers	0.938	0.699	0.239	0.000
Provider directories	Control	0.947	0.863	0.085	0.010
	Poor Oral Health Effects	0.928	0.773	0.154	0.000
	Burdens on Beneficiaries	0.962	0.871	0.091	0.003
	Poverty	0.980	0.911	0.069	0.002
	Poverty & Racial Disparities	0.895	0.906	−0.011	0.761
	Burdens on Providers	0.929	0.821	0.107	0.005
Direct care	Control	0.933	0.849	0.084	0.031
	Poor Oral Health Effects	0.970	0.815	0.156	0.000
	Burdens on Beneficiaries	0.950	0.855	0.096	0.001
	Poverty	0.952	0.914	0.038	0.158
	Poverty & Racial Disparities	0.919	0.860	0.059	0.094
	Burdens on Providers	0.954	0.782	0.172	0.000
Routine care	Control	0.876	0.817	0.059	0.174
	Poor Oral Health Effects	0.926	0.830	0.096	0.007
	Burdens on Beneficiaries	0.931	0.817	0.113	0.001
	Poverty	0.922	0.866	0.056	0.098
	Poverty & Racial Disparities	0.905	0.819	0.086	0.020
	Burdens on Providers	0.949	0.831	0.118	0.000
Credentials	Control	0.934	0.850	0.084	0.026
	Poor Oral Health Effects	0.980	0.874	0.106	0.000
	Burdens on Beneficiaries	0.963	0.903	0.060	0.018
	Poverty	0.986	0.919	0.066	0.001
	Poverty & Racial Disparities	0.924	0.919	0.005	0.869
	Burdens on Providers	0.927	0.835	0.092	0.015
Reimbursement	Control	0.923	0.885	0.038	0.255
	Poor Oral Health Effects	0.955	0.853	0.102	0.002
	Burdens on Beneficiaries	0.932	0.896	0.036	0.283
	Poverty	0.962	0.916	0.046	0.078
	Poverty & Racial Disparities	0.931	0.898	0.033	0.287
	Burdens on Providers	0.928	0.896	0.032	0.325

Policy	Treatment	Low racial resentment	High racial resentment	Delta	*p*
Referral	Control	0.807	0.619	0.188	0.000
	Poor Oral Health Effects	0.830	0.561	0.270	0.000
	Burdens on Beneficiaries	0.818	0.556	0.261	0.000
	Poverty	0.870	0.643	0.227	0.000
	Poverty & Racial Disparities	0.842	0.668	0.173	0.000
	Burdens on Providers	0.844	0.653	0.191	0.000

Note: Based on a national survey of 5784 U.S. residents from May 7 to 15, 2024. Respondents were offered the binary choices whether states should implement the burden-reducing policies. Higher probabilities indicate higher levels of support for implementing the policy. For details on policy choices refer to text and the appendix.

Appendix L

Comparison Across by Connection to Medicaid Program

Policy	Treatment	Low racial resentment	High racial resentment	Delta	*p*
Outreach	Control	0.906	0.760	0.146	0.000
	Poor Oral Health Effects	0.901	0.778	0.123	0.000
	Burdens on Beneficiaries	0.893	0.740	0.153	0.000
	Poverty	0.913	0.821	0.092	0.001
	Poverty & Racial Disparities	0.911	0.816	0.094	0.000
	Burdens on Providers	0.900	0.795	0.105	0.001
Provider directories	Control	0.961	0.901	0.061	0.004
	Poor Oral Health Effects	0.886	0.860	0.026	0.401
	Burdens on Beneficiaries	0.959	0.890	0.069	0.002
	Poverty	0.951	0.935	0.017	0.399
	Poverty & Racial Disparities	0.951	0.874	0.077	0.001
	Burdens on Providers	0.941	0.879	0.062	0.011
Direct care	Control	0.917	0.903	0.014	0.594
	Poor Oral Health Effects	0.925	0.870	0.056	0.041
	Burdens on Beneficiaries	0.939	0.901	0.038	0.064
	Poverty	0.955	0.908	0.047	0.016
	Poverty & Racial Disparities	0.946	0.881	0.065	0.004
	Burdens on Providers	0.921	0.859	0.062	0.025
Routine care	Control	0.904	0.864	0.040	0.157
	Poor Oral Health Effects	0.907	0.845	0.062	0.028
	Burdens on Beneficiaries	0.910	0.851	0.059	0.028
	Poverty	0.911	0.869	0.042	0.100
	Poverty & Racial Disparities	0.922	0.860	0.062	0.008
	Burdens on Providers	0.904	0.878	0.027	0.318

Policy	Treatment	Low racial resentment	High racial resentment	Delta	*p*
Credentials	Control	0.937	0.893	0.044	0.080
	Poor Oral Health Effects	0.940	0.909	0.030	0.189
	Burdens on Beneficiaries	0.934	0.924	0.010	0.605
	Poverty	0.978	0.917	0.062	0.000
	Poverty & Racial Disparities	0.959	0.893	0.066	0.001
	Burdens on Providers	0.919	0.897	0.022	0.405
Reimbursement	Control	0.915	0.903	0.012	0.626
	Poor Oral Health Effects	0.926	0.885	0.041	0.112
	Burdens on Beneficiaries	0.915	0.906	0.009	0.719
	Poverty	0.948	0.918	0.030	0.151
	Poverty & Racial Disparities	0.967	0.883	0.083	0.000
	Burdens on Providers	0.935	0.910	0.025	0.236
Referral	Control	0.805	0.703	0.102	0.003
	Poor Oral Health Effects	0.798	0.677	0.121	0.001
	Burdens on Beneficiaries	0.762	0.646	0.117	0.002
	Poverty	0.801	0.716	0.085	0.018
	Poverty & Racial Disparities	0.819	0.700	0.119	0.000
	Burdens on Providers	0.815	0.719	0.095	0.004

Note: Based on a national survey of 5784 U.S. residents from May 7 to 15, 2024. Respondents were offered the binary choices whether states should implement the burden-reducing policies. Higher probabilities indicate higher levels of support for implementing the policy. For details on policy choices refer to text and the appendix.

Appendix M

Comparison Across Levels of Burden Tolerance

Policy	Treatment	Low racial resentment	High racial resentment	Delta	*p*
Outreach	Control	0.894	0.723	0.171	0.000
	Poor Oral Health Effects	0.873	0.772	0.101	0.009
	Burdens on Beneficiaries	0.855	0.773	0.082	0.030
	Poverty	0.899	0.818	0.080	0.011
	Poverty & Racial Disparities	0.897	0.794	0.103	0.003
	Burdens on Providers	0.868	0.766	0.101	0.010
Provider directories	Control	0.971	0.869	0.102	0.000
	Poor Oral Health Effects	0.910	0.839	0.071	0.054
	Burdens on Beneficiaries	0.955	0.883	0.072	0.011
	Poverty	0.981	0.915	0.066	0.001
	Poverty & Racial Disparities	0.952	0.882	0.070	0.016
	Burdens on Providers	0.931	0.841	0.090	0.006

Policy	Treatment	Low racial resentment	High racial resentment	Delta	*p*
Direct care	Control	0.931	0.876	0.055	0.083
	Poor Oral Health Effects	0.941	0.844	0.097	0.002
	Burdens on Beneficiaries	0.969	0.888	0.081	0.001
	Poverty	0.933	0.910	0.023	0.365
	Poverty & Racial Disparities	0.944	0.852	0.093	0.002
	Burdens on Providers	0.919	0.803	0.115	0.002
Routine care	Control	0.899	0.862	0.037	0.276
	Poor Oral Health Effects	0.917	0.862	0.055	0.084
	Burdens on Beneficiaries	0.936	0.860	0.076	0.007
	Poverty	0.919	0.857	0.063	0.035
	Poverty & Racial Disparities	0.921	0.861	0.060	0.037
	Burdens on Providers	0.921	0.836	0.086	0.010
Credentials	Control	0.953	0.854	0.098	0.002
	Poor Oral Health Effects	0.962	0.896	0.066	0.016
	Burdens on Beneficiaries	0.952	0.900	0.052	0.035
	Poverty	0.970	0.935	0.035	0.067
	Poverty & Racial Disparities	0.971	0.903	0.068	0.005
	Burdens on Providers	0.933	0.831	0.102	0.003
Reimbursement	Control	0.955	0.858	0.097	0.002
	Poor Oral Health Effects	0.935	0.872	0.064	0.051
	Burdens on Beneficiaries	0.942	0.871	0.071	0.025
	Poverty	0.957	0.907	0.049	0.056
	Poverty & Racial Disparities	0.963	0.896	0.068	0.008
	Burdens on Providers	0.962	0.877	0.084	0.001
Referral	Control	0.773	0.711	0.062	0.159
	Poor Oral Health Effects	0.717	0.739	−0.023	0.608
	Burdens on Beneficiaries	0.707	0.684	0.023	0.608
	Poverty	0.760	0.751	0.008	0.838
	Poverty & Racial Disparities	0.764	0.727	0.037	0.362
	Burdens on Providers	0.798	0.719	0.079	0.049

Note: Based on a national survey of 5784 U.S. residents from May 7 to 15, 2024. Respondents were offered the binary choices whether states should implement the burden-reducing policies. Higher probabilities indicate higher levels of support for implementing the policy. For details on policy choices refer to text and the appendix.

Appendix N

Comparison Across Levels of Administrative Capital

Policy	Treatment	Low racial resentment	High racial resentment	Delta	*p*
Outreach	Control	0.846	0.783	0.062	0.121
	Poor Oral Health Effects	0.851	0.809	0.042	0.280
	Burdens on Beneficiaries	0.871	0.748	0.124	0.001
	Poverty	0.887	0.829	0.058	0.071
	Poverty & Racial Disparities	0.916	0.825	0.091	0.001
	Burdens on Providers	0.888	0.800	0.088	0.021
Provider directories	Control	0.947	0.910	0.037	0.129
	Poor Oral Health Effects	0.868	0.892	−0.024	0.519
	Burdens on Beneficiaries	0.911	0.934	−0.023	0.449
	Poverty	0.949	0.934	0.015	0.471
	Poverty & Racial Disparities	0.916	0.920	−0.004	0.890
	Burdens on Providers	0.949	0.879	0.070	0.014
Direct care	Control	0.922	0.912	0.010	0.732
	Poor Oral Health Effects	0.923	0.883	0.040	0.185
	Burdens on Beneficiaries	0.941	0.909	0.032	0.156
	Poverty	0.920	0.938	−0.018	0.457
	Poverty & Racial Disparities	0.924	0.912	0.012	0.650
	Burdens on Providers	0.954	0.854	0.100	0.000
Routine care	Control	0.927	0.883	0.044	0.100
	Poor Oral Health Effects	0.892	0.876	0.016	0.637
	Burdens on Beneficiaries	0.910	0.871	0.039	0.169
	Poverty	0.898	0.873	0.025	0.419
	Poverty & Racialw Disparities	0.930	0.883	0.047	0.077
	Burdens on Providers	0.913	0.878	0.035	0.279
Credentials	Control	0.949	0.905	0.045	0.065
	Poor Oral Health Effects	0.929	0.937	−0.008	0.761
	Burdens on Beneficiaries	0.925	0.932	−0.007	0.753
	Poverty	0.949	0.938	0.011	0.617
	Poverty & Racial Disparities	0.945	0.926	0.020	0.361
	Burdens on Providers	0.944	0.893	0.052	0.080
Reimbursement	Control	0.950	0.908	0.042	0.078
	Poor Oral Health Effects	0.869	0.945	−0.077	0.030
	Burdens on Beneficiaries	0.924	0.937	−0.014	0.595
	Poverty	0.911	0.943	−0.032	0.241
	Poverty & Racial Disparities	0.945	0.927	0.018	0.384
	Burdens on Providers	0.937	0.915	0.022	0.397

Policy	Treatment	Low racial resentment	High racial resentment	Delta	*p*
Referral	Control	0.791	0.673	0.118	0.006
	Poor Oral Health Effects	0.784	0.687	0.097	0.028
	Burdens on Beneficiaries	0.701	0.653	0.048	0.316
	Poverty	0.809	0.698	0.110	0.006
	Poverty & Racial Disparities	0.807	0.721	0.086	0.028
	Burdens on Providers	0.791	0.729	0.062	0.146

Note: Based on a national survey of 5784 U.S. residents from May 7 to 15, 2024. Respondents were offered the binary choices whether states should implement the burden-reducing policies. Higher probabilities indicate higher levels of support for implementing the policy. For details on policy choices refer to text and the appendix.Supporting Information

Additional supporting information can be found online in the Supporting Information section. **Table S3:** Model predicting the odds of intending to stay with inclusion of all organizational factors. **Table S4:** Model predicting the odds of intending to stay with inclusion of all individual motivators. **Table S5:** Model predicting the odds of intending to stay with inclusion of all individual perceptions. **Table S6:** Model predicting the odds of intending to stay with inclusion of all individual characteristics.
